# Editorial: Beta cell function and diabetes remission

**DOI:** 10.3389/fendo.2023.1298101

**Published:** 2023-12-15

**Authors:** Xiaopei Cao, Nan Chen, Yanbing Li

**Affiliations:** Department of Endocrinology, First Affiliatted Hospital, Sun Yat-sen University, Guangzhou, China

**Keywords:** type 2 diabetes mellitus, type 2 diabetes remission, beta-cell function, beta-cell reserve, mechanism

Type 2 diabetes (T2D) develops when insulin secretion is insufficient to meet the requirements of an individual’s insulin resistance. Recent studies have reported that some patients with T2D can achieve a durable normal glucose state through lifestyle modification, metabolic surgery, or short-term intensive therapeutic interventions (1–3). This phenomenon is described as type 2 diabetes remission and is defined as having an HbA1c of below 6.5% for a period of time in the absence of glucose-lowering pharmacotherapy.

Islet beta-cell capacity to recover is critical for T2D remission. This Research Topic focuses on beta-cell function and diabetes remission to understand the underlying mechanisms. The present Research Topic assembles four original research papers that demonstrate the status of beta-cell research, the mechanism of beta-cell dysfunction, and related clinical research.

T2D remission can be achieved through lifestyle changes as well as medical interventions such as intensive insulin therapy and bariatric surgery. Weir and Bonner-Weir. pointed out that the major driver for the above strategies for obtaining diabetes remission is the reduction of glucose levels. Increased glucose levels have a toxic effect on beta cells, defined as glucose toxicity, which exerts a profound inhibitory effect on insulin secretion. This beta-cell dysfunction can be reversed by therapies that reduce glucose levels, providing a strong rational for aggressive glucose-lowering treatments for diabetes. In addition to the role of glucose toxicity, the twin cycle hypothesis proposed by Taylor highlights that excess intra-organ fat accumulation could potentially explain liver and pancreas dysfunction (4). Excess fat removal from these organs by significant weight loss was associated with beta-cell recovery and improved acute insulin secretion in response glucose challenge and with sustained remission (5). Understanding the toxic effects of glucose and lipids on β cells has been a focus of research for decades. However, whether intrapancreatic fat directly affects β-cell function remains an important question yet to be answered. Although β-cell lipotoxicity has been convincingly proven in animal models and *in vitro* cell studies, its role in β-cell dysfunction in human diabetes *in vivo* remains a subject of debate and remains to be unveiled (6).

Understanding remission requires knowledge of the mechanisms of beta-cell dysfunction. The pathophysiological processes and recovery of beta-cell dysfunction have been extensively studied. Luo et al. conducted a pioneering study utilizing bibliometric and knowledge-map approaches to present a comprehensive overview of beta-cell research. The number of publications on beta-cell exhibited a steady increase over time. They found that current studies on beta cells primarily focused on the pathogenesis and risk factors of beta-cell failure, as well as strategies for the restoration of beta cells. How to restore beta-cell mass and function will continue to be a focus in the future. Apoptosis, oxidative stress, and endoplasmic reticulum stress have been identified as mechanisms underlying β-cell failure over the past two decades. In the recent decade, research has also focused on novel mechanisms such as miRNA, autophagy, dedifferentiation, and inflammation to further understand β-cell failure. This valuable information will provide an essential reference for researchers specializing in β-cell research. In addition to the above-mentioned factors, ferroptosis has been found to be a novel factor in pancreatic beta-cell death in diabetes. Iron accumulation, observed in type 2 diabetic islet beta cells, is associated with insulin secretion dysfunction in pancreatic beta cells (7). Qin et al. conducted a clinical study and showed that serum ferritin levels were significantly increased in Chinese patients with newly diagnosed T2DM compared to healthy control subjects. In addition, serum ferritin was an independent risk factor associated with impaired beta-cell function, and transferrin was an independent protective factor against beta-cell functional impairment in male patients.

Diabetes remission is dependent on the reversibility of islet beta-cell dysfunction. There is growing evidence that beta-cell dedifferentiation and reprogramming play a significant role in the regulation of beta-cell function in the early and middle stages of T2D progression, which serve as the basis for restoring beta-cell function (8, 9). Therefore, among the mechanisms mentioned above, beta cell dedifferentiation deserves special attention to understand remission.

This Research Topic also includes an article about the relationship between insulin resistance and T2DM-related renal complications. Gao et al. report the results of a single-center retrospective cohort study that found a positive association between the baseline triglyceride-glucose index, a surrogate marker of insulin resistance, and the risk of future end-stage renal disease (ESRD) in patients with T2D and chronic kidney disease. This association will need to be validated in future studies with a larger sample size and a longer follow-up time. The correlation between the triglyceride-glucose index and diabetes complications also suggests that reducing insulin resistance through glucose and lipid reduction is associated with the prevention or delay of diabetes complications. The mechanisms involved in diabetes remission also apply to the prevention of diabetes complications.

We commend the authors for their work and their contributions. In conclusion, realizing the reversibility of beta-cell dysfunction and further exploring the underlying mechanisms of T2D will promote the study of diabetes remission.

## Author contributions

XC: Writing – original draft, Writing – review & editing. NC: Writing – original draft. YL: Writing – review & editing.
